# Diterpenoid alkaloids of *Aconitum laciniatum* and mitigation of inflammation by 14-*O*-acetylneoline in a murine model of ulcerative colitis

**DOI:** 10.1038/srep12845

**Published:** 2015-08-04

**Authors:** Phurpa Wangchuk, Severine Navarro, Catherine Shepherd, Paul A. Keller, Stephen G. Pyne, Alex Loukas

**Affiliations:** 1 Centre for Biodiscovery and Molecular Development of Therapeutics, Australian Institute of Tropical Health and Medicine, James Cook University, Cairns, QLD, Australia; 2 School of Chemistry, University of Wollongong, Wollongong, NSW, Australia

## Abstract

*Aconitum laciniatum* is used in Bhutanese traditional medicine for treating various chronic infections and inflammatory conditions. We carried out in-depth isolation and characterization of the phytochemicals from the root component and determined the anti-inflammatory effects of the isolated compounds against chemically-induced colitis in mice. Five diterpenoid alkaloids - pseudaconitine, 14-veratroylpseudaconine, 14-*O*-acetylneoline, neoline, and senbusine A - were isolated from *A. laciniatum* for the first time. Two of the alkaloids were tested for anti-inflammatory properties in the TNBS-induced colitis model in mice. Various parameters were measured to assess pathology including weight loss, clinical and macroscopic scores, histological structure and IFN-γ production in the gut. Of the two alkaloids tested, 14-*O*-acetylneoline showed significant protection against different parameters of colitic inflammation. Compared to control mice that received TNBS alone, mice treated with 14-*O*-acetylneoline experienced significantly less weight loss and had significantly lower clinical scores, macroscopic pathology and grades of histological inflammation. Moreover, colonic IFN-γ mRNA levels were significantly reduced in mice that received 14-*O*-acetylneoline compared to control mice that received TNBS alone. This alkaloid is now considered a novel anti-colitis drug lead compound.

*Aconitum laciniatum* (Ranunculaceae) is one of the 100 species of aconites reported from the temperate and alpine regions of the northern hemisphere, including Bhutan^1^. Traditionally, aconites have been used in Asia, Alaska, and Europe for a wide range of applications ranging from warfare, hunting, covert human poisons and in traditional medicines^2^. In European homeopathy and Asian traditional medicines, many species of aconites are used for treating various ailments, particularly those with an inflammatory basis^3^. In Bhutanese traditional medicine (BTM), of the 19 *Aconitum* species reported^4^, three of them including *A. laciniatum, A. violaceum* and *A. orochryseum* are used in the preparation of 25 polyherbal formulations for treating numerous disorders including inflammatory conditions^1^,^5^. Many *Aconitum* species are poisonous due to their rich content of diterpenoid alkaloids and need to be pre-processed and detoxified prior to their use^6^,^7^,^8^. However, carefully defined doses have been demonstrated to contribute to the desired clinical effects^8^,^9^,^10^. In modern biomedicine, aconite-containing ointments, including Aconitysat^TM^, Brinpax^TM^, Etermol^TM^ and Pectovox^TM^, are used as anodynes in the treatment of chronic rheumatism, neuralgia and sciatica^11^,^12^.

Phytochemically, more than 197 diterpenoid alkaloids (DA) with complicated structures have been isolated from 56 *Aconitum* species of pharmacological significance^13^,^14^,^15^. These *Aconitum* DA have been reported to possess a broad range of bioactivities including anti-inflammatory properties^3^,^9^,^16^,^17^,^18^,^19^,^20^,^21^.

All inflammatory conditions, especially the inflammatory bowel diseases (IBD), are chronic immune-mediated diseases with multi-factorial aetiologies influenced by genetics, environment, diet and the composition of the gut microbiota^22^. The prevalence of IBD is rising dramatically in developed countries, and populations previously considered to be low risk (such as in Japan and India) are now experiencing an increase in the incidence of IBD^23^. Current treatment for IBD includes corticosteroids and biologics such as monoclonal antibodies targeting Tumour Necrosis Factor (TNF), but these drugs manage the disease rather than cure it, and drug failure rates are high^24^, precipitating the need for new approaches to treat IBD. A recent review on herbal and plant therapies for the treatment of human IBD^25^ noted that 27 clinical studies have been conducted by administering 11 herbal/plant therapies to a total of 1,874 IBD patients. Of the 11 plant samples, seven of them were reported to have beneficial effects on ulcerative colitis, while four plants resulted in clinical remission of Crohn’s disease.

In this study, we explored one of the Bhutanese medicinal plants, *A. laciniatum*, which is traditionally used for treating chronic parasitic and microbial infections, as well as inflammatory conditions^26^. Its flowers, leaves and roots are collected annually for formulating at least five important polyherbal BTM formulations^5^. Our earlier work on the crude methanol extract of this plant showed partial inhibition of the production of the pro-inflammatory cytokine, TNF-α by LPS-activated monocytes^27^. Directed by this finding and its ethnopharmacological uses, we carried out in-depth isolation and characterization of the phytochemicals and determined the anti-inflammatory effects of the isolated compounds against chemically-induced colitis in mice.

## Results

In the current study, we isolated, for the first time from *A. laciniatum*, five diterpenoid alkaloids and showed that one of the compounds protected mice against inflammation in the trinitrobenzoylsulfonic acid (TNBS)-induced mouse model of colitis that shares many key features with human ulcerative colitis.

### Phytochemical isolation and characterization

**Figure 1:**
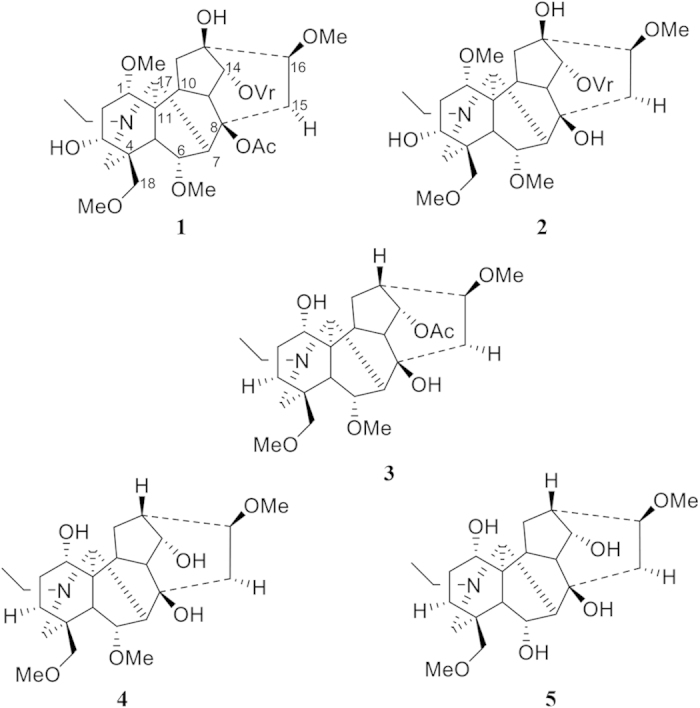
C_19_-diterpenoid alkaloids isolated from *A. laciniatum* (1-5). pseudaconitine (**1**), 14-veratroylpseudaconine (**2**), 14-*O*-acetylneoline (**3**), neoline (**4**), senbusine A (**5**).

About 165 g of crude MeOH extract was obtained from two kilograms of dry powdered roots of *A. laciniatum* upon extraction with MeOH giving an extractive value of 8.25% (w/w). Through an acid-base alkaloid isolation method, the DCM-1 and DCM-2 extracts were obtained. We repeatedly subjected these extracts to the silica gel column and preparatory thin layer chromatography separation protocols to obtain five C_19_-diterpenoid alkaloids (Fig. 1). Initial purification of DCM-1 fraction yielded compounds **1** and **2** (Fig. 1). Compound **1** (1.9 g) was identified as the major chemical constituents of the plant. Its LR-ESI-MS showed a [M + H^+^] peak at (*m/z*): 690 and the LR-EI-MS ion fragmentation pattern and the ^1^H-NMR spectroscopic data of this alkaloid (**1**) agreed well with those of pseudaconitine reported in the literature^28^. Cross-examination of the published literature found that this compound was isolated earlier from the roots of other aconites^29^. Compound **2** was isolated as an amorphous creamy white solid and its LR-ESI-MS displayed the [M + H^+^] peak at (*m/z*): 648 corresponding to the molecular formula of C_34_H_49_NO_11_ (M + H^+^, calculated for 648.3384). Its ^1^H-NMR spectroscopic data agreed with those of 14-veratroylpseudaconine reported from *A. ferox*^30^ and *A. falconeri*^29^. From another fraction DCM-2, compounds **3**, **4** and **5** were obtained (Fig. 1). Compound **3** was isolated as an amorphous creamy white solid. Its LR-ESI-MS showed a [M + H^+^] peak at (*m/z*): 480 and the HR-EI-MS showed a peak at 480.2955 corresponding to the protonated molecular formula C_26_H_41_NO_7_. The ^1^H-NMR and ^13^C-NMR spectroscopic data agreed with those of 14-*O*-acetylneoline isolated from the aphids *Brachycaudus aconite*, which lives and feeds on *A. napellus*^31^. Compound **4** was isolated as an amorphous powder and its LR-ESI-MS showed a [M + H^+^] peak at (*m/z*): 438 and the HR-EI-MS exhibited a peak at 438.2863 which corresponded to the protonated molecular formula of C_24_H_39_NO_6_. These ^1^H-NMR spectroscopic data agreed with those of neoline that was isolated previously from *A. ferox*^28^. Compound **5** was isolated as an amorphous powder and its LR-ESI-MS showed a [M + H^+^] peak at (*m/z*): 424. The ^1^H-NMR as well as its APT spectroscopic data agreed with those of senbusine A isolated from *A. carmichaeli* and *A. ferox*^28^,^32^. To the best of our knowledge, compounds **2** and **3** have never been tested for any biological activities.

### Preliminary assessment of 14-veratroylpseudaconine and 14-*O*-acetylneoline for their anti-inflammatory activities in the TNBS-induced colitis mouse model

We carried out preliminary screening of compounds **2** and **3** (14-veratroylpseudaconine and 14-*O*-acetylneoline) using the TNBS-induced colitis model in mice^33^,^34^,^35^, monitoring just weight loss initially (data not shown). 14-Veratroylpseudaconine exacerbated the colitic inflammation induced by TNBS, however 14-*O*-acetylneoline induced significant protection against TNBS-induced weight loss. Further studies in the colitis model were therefore restricted to just 14-*O*-acetylneoline. Upon administration of 14-*O*-acetylneoline at doses of 10, 20, and 50 μg per mouse, mice were monitored and scored daily for mortality and clinical signs including changes in weight loss, mobility, piloerection and faecal consistency. At the end of the trial macroscopic pathology, histological structure and IFN-γ production in the gut of the mice were evaluated.

### Treatment with 14-*O*-acetylneoline protected mice from weight loss and improved clinical outcome

**Figure 2:**
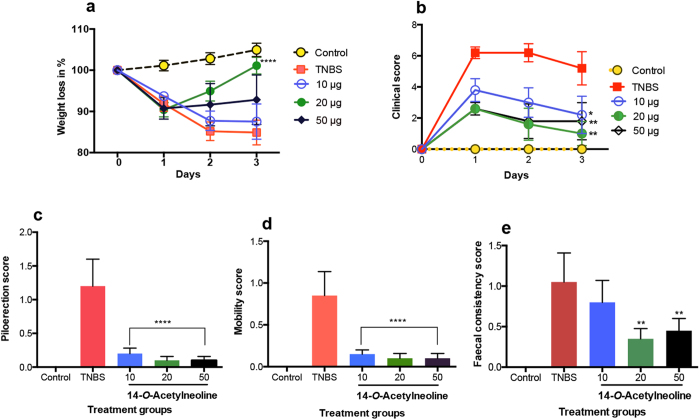
Protective effects of 14-*O-*acetylneoline against weight loss and clinical symptoms induced by TNBS colitis. (**a**) The 20 μg dose of 14-*O-*acetylneoline yielded highly significant (****P < 0.0001, 2-way ANOVA) protection against weight loss over the three-day duration of the experiment. (**b**) All three doses of 14-*O-*acetylneoline resulted in significantly reduced clinical scores in a dose-dependent manner over the three days of the experiment (*P = 0.0303 for 10 μg dose, *P = 0.0017 for 20 μg dose, **P = 0.0037 for 50 μg dose). (**c**) All three doses of 14-*O-*acetylneoline resulted in significantly reduced TNBS-induced piloerection in mice (0-2 scoring matrix) (****P < 0.0001). (**d**) All three doses of 14-*O-*acetylneoline resulted in significant protection against TNBS-induced reductions in mobility (0-3 scoring matrix) (****P < 0.0001). (**e**) The 20 and 50 μg doses of 14-*O-*acetylneoline resulted in significantly improved faecal consistency after TNBS administration. Faecal scores (0-2 scoring matrix) (**P = 0.0011 for the 20 μg dose and P = 0.0061 for the 50 μg dose). Statistical analyses were performed by pooling data from groups of mice from two independent but reproducible experiments.

Upon TNBS administration, all mice lost weight within the first 24 hours. However, mice treated with different doses (10, 20, and 50 μg) of 14-*O*-acetylneoline began to gain weight in a dose dependent manner from day 2 (Fig. 2a). Among the three doses studied, the 20 μg dose of 14-*O*-acetylneoline showed the best protective effects in terms of overall clinical scores (P = 0.0001) on day 3 compared to the TNBS-only group (Fig. 2b). All three doses of 14-*O*-acetylneoline conferred highly significant protection against pilorerection (Fig. 2c) and mobility (Fig. 2d) compared to the TNBS-only group. When faecal consistency was measured, the 20 and 50 μg doses of 14-*O*-acetylneoline resulted in significantly improved faecal consistency compared to the TNBS-only group (Fig. 2e).

### Reduced pathological changes after administration of 14-*O*-acetylneoline

**Figure 3:**
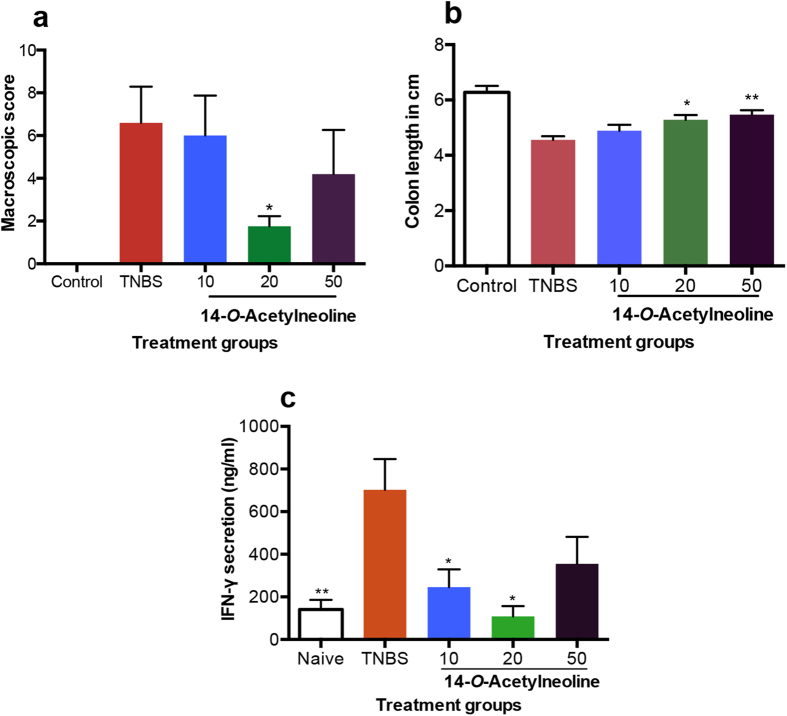
Administration of 14-*O*-acetylneoline protects mice against TNBS-induced pathological changes, colon shortening and colonic IFN-γ production. (**a**) Dose 20 μg of this compound reduced macroscopic scores over the three days of the experiment as compared to TNBS (*P < 0.0476). (**b**) Doses 20 (*P = 0.0160) and 50 μg (**P = 0.0023) of the same compound also reduced colon shortening as compared to TNBS. (c) Doses 10 and 20 significantly reduced IFN-γ production in mice as compared to TNBS (*P = 0.0242 for 20 μg dose, *P = 0.0271 for 10 μg dose, **P = 0.0011 for Naive). IFN-γ was measured using sandwiched ELISA. Statistical analyses were performed by pooling data from groups of mice from two independent but reproducible experiments.

On day 3, mice were euthanized and the colons were removed and assessed for changes in macroscopic appearances including adhesion, bowel wall thickening, mucosal oedema, ulceration, necrosis, and colon length. Mice treated with the 20 μg dose of 14-*O*-acetylneoline had significantly reduced pathology scores (Fig. 3a) (P = 0.0476) and longer colon lengths (Fig. 3b) (P = 0.0160) compared to TNBS-only mice. We observed a trend towards reduced macroscopic pathology in colon tissues of mice treated with the 50 μg dose of 14-*O*-acetylneoline compared to TNBS-only controls, but the change did not reach significance. Colon lengths of mice treated with 50 μg 14-*O*-acetylneoline were however significantly longer than those of TNBS-only mice (P = 0.0023).

### Inhibition of colonic IFN-γ production by 14-*O*-acetylneoline

The clinical and macroscopic pathology scores were consistent with reduced production of IFN-γ in the colon tissues of mice that received 14-*O*-acetylneoline in a dose dependent manner. While all doses resulted in reduced IFN-γ production, mice that received 10 μg (P = 0.0271) and 20 μg (P = 0.0242) doses exhibited significantly lower IFN-γ levels than TNBS-only mice group (Fig. 3c). The naive group, as expected, showed the lowest IFN-γ levels (P = 0.0011). Mice that received the 50 μg dose also showed lower levels of IFN-γ than TNBS-only mice, although the reduction was not significant.

### Histological analysis reveals protection of colon from TNBS-induced inflammation

**Figure 4:**
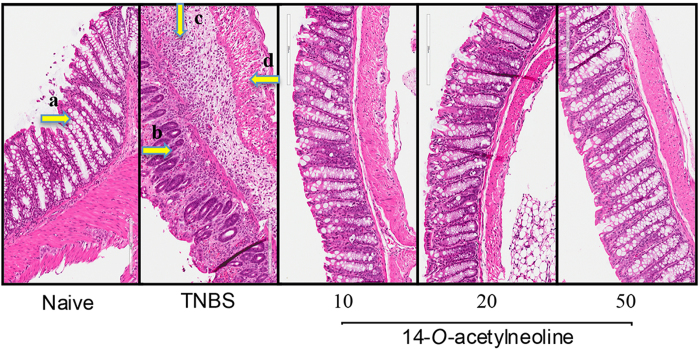
Administration of 14-*O-*acetylneoline protects mice against TNBS-induced colonic inflammation. Histological photomicrographs of hematoxylin and eosin-stained (H/E) paraffin sections (x200) of distal colon tissues, which were obtained from the representative groups of healthy mice (Naive) and mice that received TNBS and different doses of 14-*O-*acetylneoline. (**a**) Healthy goblet cells in Naive group, which can be also observed well preserved in the colon tissues of mice, treated with different doses of the compound. Destroyed goblets cells (**b**), erosion and thickening of lamina propria (**c**), and colon wall thickening (**d)** were observed in TNBS group.

The H/E stained paraffin-embedded colon sections were observed for histological changes induced by TNBS administration. Colons from the untreated naive group showed normal tissue architecture with a healthy number of goblet cells (Fig. 4-Naive). The colons of TNBS-only mice had severe lesions and extensive damage to the gastric mucosa as shown by the histological photomicrographs (Fig. 4-TNBS). This group showed mucosal congestion and erosion, inflammatory cell infiltrate, and high levels of leukocyte and polymorphonuclear cell infiltration in the lamina propria, intraepithelial compartments, and colon wall with evidence of oedema, destruction and loss of healthy goblet cells; and thickening of the lamina propria and colon wall. None of the tissues treated with different doses of 14-*O*-acetylneoline showed the characteristic histological inflammation of the TNBS-only group. All doses reduced the area of ulceration and the level of leukocyte and polymorphonuclear cell infiltration. The general appearance of the histological architecture including lamina propria, epithelial compartments, goblet cell content, and colonic wall/musculature were unaffected and could not be distinguished from the naive group.

## Discussion

Aconites are poisonous plants that require care and caution. It is noteworthy that, prior to their use in traditional medicines including BTM, aconite components are detoxified using region-specific traditional approaches, all of which are highly effective at reducing the toxicity of the plant by converting its diester diterpene alkaloids to their less toxic monoester diterpene forms^10^. Several publications^8^,^9^,^10^ have suggested that carefully defined amounts of toxic aconite diterpenoid alkaloids contribute to a diverse range of desired clinical effects, and it is essential to define the dose range that distinguishes toxicity from therapeutic efficacy. For example, atisinium chloride isolated from the Bhutanese medicinal plant, *A. orochryseum*, showed potent antimalarial activity against multi-drug resistant *Plasmodium falciparum* strains, K1CB1 and TM4/8.2^20^, and 1-*O*-benzoylnapelline, a derivative of napelline isolated from the *Aconitum* species, showed antiarrhythmic activity that markedly exceeded that of the reference Class I antiarrhythmic drugs, novocainamide, quinidine and lidocaine^17^. *A. laciniatum* is traditionally indicated in BTM for treating disorders whose symptoms bare relevance to inflammatory conditions. Interestingly, our earlier study on the crude MeOH extract of *A. laciniatum*^27^ demonstrated its anti-inflammatory potential, and our work described herein suggests that 14-*O-*acetylneoline is responsible for at least some of this anti-inflammatory bioactivity.

Ulcerative colitis is a chronic immune mediated disease with multi-factorial aetiology influenced by genetics, environment and the composition of the gut microbiota^22^,^35^. Its prevalence is rising in developed countries, with North America and Europe experiencing the steepest increases in incidence over the past few decades. Moreover, populations previously considered to be low risk (such as in Japan and India) are now experiencing an increase in the incidence of IBD, especially ulcerative colitis^23^. Current treatment for IBD includes 5-aminosalicylic acid, corticosteroids and biologics, each of which has drawbacks and limitations in efficacy^36^. Traditional medicinal and dietary plants are known to have anti-inflammatory properties, especially the dietary plants due to their relative non-toxicity. Despite their increasing use by IBD patients, a lack of understanding of the bioactive chemical components and mechanisms of anti-inflammatory action is an obstacle to the incorporation of many herbal or dietary treatments into mainstream medicine. A small number of studies have confirmed that medicinal and dietary plant extracts containing terpenoids, alkaloids, flavonoids and anthocyanins exert their anti-inflammatory activities by controlling the levels of various inflammatory cytokines^37^,^38^. For example, anthocyanin-rich fractions of blueberry and raspberries attenuate inflammation in TNBS-induced colitis by suppressing inflammatory cytokine production in macrophages^39^,^40^. Curcumin obtained from *Curcuma longa* showed potent anti-inflammatory activity in TNBS-induced colitis as well as in patients with uncreative colitis without side effects or toxicity^25^,^41^.

The gastrointestinal tract contains a mucus layer that serves as the front line of innate host defence, and its breakdown can result in colitis^42^. Our histological findings demonstrate that the administration of 14-*O*-acetylneoline prevented intestinal goblet cells from degenerating as a result of TNBS-induced biochemical processes. Dysregulation of this intestinal goblet cell barrier allows the initiation of chronic inflammation that regulates the production of various mediators or proinflammatory cytokines including TNF-α and IFN-γ^43^,^44^,^45^. Both cytokines are the known intestinal signatures of IBD^46^,^47^. In our earlier findings, the MeOH extract of *A. laciniatum* partially reduced the levels of TNF-α production in LPS-activated THP-1 monocytic cells^27^. Our findings herein using an animal model of colitis have shown that 14-*O*-acetylneoline isolated from *A. laciniatum* MeOH extract significantly reduced the production of IFN-γ by mouse colonic tissues *in vivo*. Although, TNF-α was not assessed in this study, it is likely that 14-*O*-acetylneoline is the active compound within the crude MeOH extract that inhibits production of this cytokine, although further testing is required.

In conclusion, we isolated five diterpenoid alkaloids from *A. laciniatum* and show that pseudaconitine is the major constituent of the plant. Of the two alkaloids tested in TNBS-induced colitis in mice, one minor compound, 14-*O*-acetylneoline showed significant anti-colitis activity. This finding corroborates the traditional claim of anti-inflammatory properties of this plant in BTM, however the mechanism by which 14-*O*-acetylneoline exerts its anti-colitic action cannot be elucidated from our current data. Future work will focus on isolation of larger quantities of this compound from *A. laciniatum* to facilitate investigation into its mechanism of action and detailed pre-clinical studies including toxicity and derivatization analyses to generate synthetic 14-*O*-acetylneoline.

## Materials and Methods

### Plant material and isolation of compounds

*A. laciniatum* is locally known as “bdud-rtsi-lo-ma” or “tsan-dug”. This plant is an erect biennial herb, of 0.6–1.5 m in height, with less deeply dissected leaves (up to 15 cm in diameter), bluish flowers widely spaced in racemes, paired conical tubers and it grows on the grassy alpine mountain slopes of Bhutan at altitudes of 3500-4570 metres above sea level^4^. For this study, only the root of the plant was collected in August 2009 from Lingshi Makhang (Altitude: 4183 m; Latitude: 27° 50′ 29.9″; Longitude: 89° 25′ 41.5″; global positioning system point number (GPSPN): 138; Site number: P138; Slope: 25°; Aspect: North-East). The herbarium voucher specimen number 93 was assigned to it and the tissue was maintained at Menjong Sorig Pharmaceuticals, Thimphu, Bhutan. The dried roots of *A. laciniatum* were powdered and extracted with analytical/HPLC grade methanol (5 × 3 L over 48 h). The extract was filtered and then concentrated using a rotary evaporator at 35–50 °C to generate the crude MeOH extract (165 g). This extract was dissolved in MeOH/water (1:9) and then acidified with 5% HCl and fractionated with CH_2_Cl_2_ to obtain the acidic fraction (coded as DCM-1, (6.2 g)). The aqueous part was basified with NH_4_OH to pH 9–12 and then fractionated with CH_2_Cl_2_ to obtain the basic extract (coded as DCM-2, (2.4 g)) followed by ethyl acetate and *n*-butanol to obtain the respective EtOAc (0.11 g) and BuOH extracts (1.7 g). Each fraction was purified repeatedly using flash column chromatography packed with Merck Kieselgel 60 PF_254_. Aluminium-backed silica plates (0.2 mm silica thickness, Merck, normal and reverse phase) were used for separating isolates of smaller quantities. UV light (short wavelength of 254 nm, long wavelength of 366 nm) was used for visualization of the separated bands on TLC. Dragendorff’s reagent was prepared^48^ for staining the TLC plate loaded with alkaloid fractions and pure compounds. Micromass Waters Platform LCZ (single quadrupole, MeOH as solvent) was used for obtaining the LR-ESI-MS. Shimadzu GCMS-QP-5050 was used for recording the LR-EI-MS by the direct insertion technique (at 70 eV). A Micromass Waters Q-ToF Ultima (quadrupole time-of-flight) mass spectrometer was used for acquiring HR-ESI-MS. A 500 MHz Varian Unity Inova, 500 MHz Varian Premium Shield (VNMRS PS 54), and 300 MHz Varian Mercury spectrometer were used for obtaining NMR spectra (^1^H-NMR, gCOSY, ^13^C-NMR, APT, gHMBC, gHSQC, and gNOESY) using deuterated solvents (CD_3_OD or CDCl_3_) depending upon the solubility of compounds. The initial purification of the DCM-1 extract using silica gel packed CC and a gradient eluent solvent system of CH_2_Cl_2_/MeOH (100 mL, v/v ratio as 0:100, 10:90, 20:80, 30:70, 50:50) yielded eight fractions, DCM-1-F1 to DCM-1-F8. Further separation of fraction DCM-1-F4 using PTLC plates with the solvent system of MeOH-CH_2_Cl_2_-NH_4_OH (2:23:6 drops) furnished a white solid, which upon crystallization with CHCl_3_/MeOH (1:1) gave prisms. This was the major compound of the plant and was identified as pseudaconitine (**1**). Separation of fraction DCM-1-F8 using the PTLC plates with the solvent system of MeOH-CH_2_Cl_2_-NH_4_OH (1:9:6 drops) yielded 14-veratroylpseudaconine (**2**). Another fraction DCM-2 was subjected to a silica gel packed CC and a gradient eluent solvent system of CH_2_Cl_2_/MeOH (100 mL, v/v ratio as 0:100, 1:99, 2:98, 4:96, 8:92, 15:85, 30:70) yielded eight fractions, DCM-2-F1 - DCM-2-F8. Fraction DCM-2-F2 when dried furnished amorphous white solid, which was identified as 14-*O*-acetylneoline (**3**). Fraction DCM-2-F3 upon drying under reduced pressure yielded neoline (**4**). Fraction DCM-2-F7 (105.0 mg) was further separated using PTLC plates with the solvent system of MeOH-CH_2_Cl_2_ (2:23), and which identified senbusine A (**5**).

### Animal ethics and experimental TNBS-induced colitis

All animal procedures were conducted in accordance with the approved guidelines of The James Cook University Animal Ethics Committee. Male C57BL/6 mice (6 weeks old) were purchased from the Animal Resources Centre (Perth, Australia) and were housed and maintained in the QTHA animal house in accordance with Australian animal rights and regulation standards. The mice (18.06–22.28 g body weight) were confined in plastic cages with free access to pelleted food and water. They were kept under a specific pathogen-free environment at regulated temperature (22 °C) and lighting (12 h cycle). Efforts were made to reduce the number of animals used in the TNBS experiment and also to minimise their suffering.

The samples (1 mg) were dissolved in 10 μL of DMSO followed by addition of DPBS (1 mL) under sterile conditions. Mice were randomly divided into four groups with five mice in each cage as: Naive, TNBS, Veratroyl (coded for 14-veratroylpseudaconine (**2**)) and Bhutamine (coded for 14-*O*-acetylneoline (**3**)). The active alkaloid 14-*O*-acetylneoline (**3**) was later subjected to dose-dependent activity analyses using mice in the following groups: Naive, TNBS, BHUT-10 (coded for 10 μg dose)/TNBS, BHUT-20 (coded for 20 μg dose)/TNBS and BHUT-50 (coded for 50 μg dose)/TNBS. As coded above, the experimental mice received the test compound doses of 10, 20 and 50 μg, respectively. These doses were prepared in DPBS to make a total injectable volume of 200 μL/mouse. Mice were administered test compounds via the intra-peritoneal (i.p.) route and left in their cages for 5–6 hours prior to administration of TNBS.

Standard TNBS colitis protocols^33^,^34^,^35^,^40^,^41^ were employed. The TNBS (positive controls), BHUT-10, BHUT-20 and BHUT-50 groups were administered TNBS intra-rectally (3 mg per mice in 45% EtOH, 100 μL/mice) to induce transient colitis. Xylazine-ketamine solution was prepared as 5 mg/kg and 50 mg/kg, respectively. Mice were anaesthetized using a 200 μL of this solution and TNBS was carefully administered intra-rectally using a SRPLO I.V. catheter (Radiopaque/ETFE, Gauge 20G × 11/4”, I.D. 0.80 × 32 mm, Terumo Corporation) lubricated with K.Y jelly. To assure the distribution of TNBS within the entire colon lumen, mice were carefully maintained at a 90° angle (head down position) for 2 mins and then returned to their cages.

### Clinical and macroscopic assessment of mice with TNBS-induced colitis

Throughout the experiment, mice were monitored daily for weight loss and changes in clinical signs for 3 days. The clinical signs of disease including changes in body weight, mobility, piloerection and faecal consistency were scored from 0–2, zero being normal and 2 being diseased. A score of “0” was given when the mice gained weight, “1” when weight remained the same and “2” upon losing weight. For mobility, a score of “0” was given for normal, “1” for movement only after stimulation, and “2” for lethargic mice that exhibited little to no movement after stimulation. Mice with no piloerection scored “0,” mild piloerection over the neck as “1” and severe piloerection all over the body as “2”. Normal faeces scored “0”, mild diarrhoea was scored “1” and severe diarrhoea with blood was scored “2”. On day 3 post-TNBS administration, the mice were euthanized and the colon harvested and then transferred to a petri dish with sterile DPBS. Tissues were opened longitudinally, washed with DPBS, placed under a microscope (Olympus SZ61, 0.67-4.5x), assessed for changes in macroscopic appearance, and then scored for pathological changes as follows: adhesion (0 to 3), bowel wall thickening (0 to 3), mucosal oedema (0 to 3), ulceration (0 to 3), and colon length as described previously^47^.

### Histological analysis and evaluation of microscopic damage

Distal colon tissue (1 cm) obtained from representative groups of healthy mice (Naive) and mice that received TNBS and different doses of 14-*O-*acetylneoline were placed into 4% paraformaldehyde (1 mL) and stored for 24 hr at 4 °C. These tissues were then transferred to EtOH (70%) prior to being embedded in paraffin. Sections were stained with haematoxylin and eosin (H/E), observed for histological changes by light microscopy and histological photomicrographs (x 200) were captured. Histological and microscopic observation included changes in overall colon structure involving inflammation, gut lining, goblets cells, lamina propria, and colon wall.

### Cell preparation and IFN-γ quantification

Colon pieces (1 cm) were collected after mice were euthanized and placed in sterile 24 well tissue culture plates with 500 μL of complete medium with foetal bovine serum (5 ml), 100 U of penicillin/ml, 100 μg of streptomycin, β-mercaptoethanol (55 μM), HEPES buffer (10 mM). Tissues were cultured for 24 hr at 37 °C after which supernatants were collected and stored at −80 °C until further use. Colon culture supernatants were thawed and IFN-γ levels quantified using a sandwich enzyme-linked immunosorbent assay (ELISA) (mouse IFN-γ ELISA Ready-SET-Go! eBiosciences) following the manufacturer’s instructions. OD_490_ were measured using a POLARstar Omega plate reader (BMG LABTECH).

### Statistical analyses

All data was analysed with GraphPad Prism (version 6.0e). Statistical analyses were performed using the 2-way ANOVA, multiple comparisons of treatment group over different time. Unpaired Mann-Whitney non-parametric tests were used for comparing pooled data from repeated experiments. All values are expressed as means ± SEM. The significance *p*-values were calculated using two-tailed tests with over 95% confidence levels. Results were considered significant when P < 0.05. Statistical analyses were performed by pooling data from groups of mice from two independent but reproducible experiments such that N = 8–10 mice per group.

## Additional Information

**How to cite this article**: Wangchuk, P. *et al.* Diterpenoid alkaloids of *Aconitum laciniatum* and mitigation of inflammation by 14-*O*-acetylneoline in a murine model of ulcerative colitis. *Sci. Rep.* **5**, 12845; doi: 10.1038/srep12845 (2015).
